# Diversity of Lysis-Resistant Bacteria and Archaea in the Polyextreme Environment of Salar de Huasco

**DOI:** 10.3389/fmicb.2022.826117

**Published:** 2022-04-25

**Authors:** Andrea Corona Ramírez, Guillaume Cailleau, Mathilda Fatton, Cristina Dorador, Pilar Junier

**Affiliations:** ^1^Laboratory of Microbiology, Institute of Biology, University of Neuchâtel, Neuchâtel, Switzerland; ^2^Department of Biotechnology, University of Antofagasta, Antofagasta, Chile

**Keywords:** lysis-resistant, lake sediment, microbial mat, extreme environment, archaea, bacteria

## Abstract

The production of specialized resting cells is a remarkable strategy developed by several organisms to survive unfavorable environmental conditions. Spores are specialized resting cells that are characterized by low to absent metabolic activity and higher resistance. Spore-like cells are known from multiple groups of bacteria, which can form spores under suboptimal growth conditions (e.g., starvation). In contrast, little is known about the production of specialized resting cells in archaea. In this study, we applied a culture-independent method that uses physical and chemical lysis, to assess the diversity of lysis-resistant bacteria and archaea and compare it to the overall prokaryotic diversity (direct DNA extraction). The diversity of lysis-resistant cells was studied in the polyextreme environment of the Salar de Huasco. The Salar de Huasco is a high-altitude athalassohaline wetland in the Chilean Altiplano. Previous studies have shown a high diversity of bacteria and archaea in the Salar de Huasco, but the diversity of lysis-resistant microorganisms has never been investigated. The underlying hypothesis was that the combination of extreme abiotic conditions might favor the production of specialized resting cells. Samples were collected from sediment cores along a saline gradient and microbial mats were collected in small surrounding ponds. A significantly different diversity and composition were found in the sediment cores or microbial mats. Furthermore, our results show a high diversity of lysis-resistant cells not only in bacteria but also in archaea. The bacterial lysis-resistant fraction was distinct in comparison to the overall community. Also, the ability to survive the lysis-resistant treatment was restricted to a few groups, including known spore-forming phyla such as Firmicutes and Actinobacteria. In contrast to bacteria, lysis resistance was widely spread in archaea, hinting at a generalized resistance to lysis, which is at least comparable to the resistance of dormant cells in bacteria. The enrichment of *Natrinema* and *Halarchaeum* in the lysis-resistant fraction could hint at the production of cyst-like cells or other resistant cells. These results can guide future studies aiming to isolate and broaden the characterization of lysis-resistant archaea.

## Introduction

Natural environments can experience fluctuation in biotic and abiotic factors, which impacts the growth and survival of the organisms living within them. In response, organisms must develop strategies to survive under environmental conditions that are suboptimal for their growth and reproduction (Yang et al., 2016; Gray et al., 2019). One common response is dormancy (Logan et al., 2000; Wood et al., 2013), which consists of the decrease in the metabolic activity of the organism and it can be achieved through different mechanisms (Jones and Lennon, 2010; Lennon and Jones, 2011; Hice et al., 2018). One of them is the production of specialized cells (Cáceres and Tessier, 2003). Single-cell or multicellular organisms can create such types of specialized structures for survival. Parasitic and free-living protists that form cysts (highly resistant structures) are examples (Aguilar-Díaz et al., 2011; Lambrecht et al., 2015). Furthermore, bacteria are known to form one of the most resistant dormant specialized structures, called endospores, which can resist physical and chemical stressors (Nicholson et al., 2000; Setlow, 2014).

Since the discovery of bacterial spores in the last quarter of the 19th century (Koch, 1876), microbiologists have uncovered many elements of the sporulation process, such as the triggers for entering sporulation and cell re-activation, levels of resistance, and structural properties of spores, among others. This has been done mainly for spores and spore-like cells from five bacterial groups: Firmicutes (Nicholson et al., 2000; Higgins and Dworkin, 2012), Actinobacteria (Chater et al., 2010; Sexton and Tocheva, 2020), Cyanobacteria (Herdman, 1988; Kaplan-Levy et al., 2010), and in the δ-proteobacterial orders *Myxococcales* (Kottel et al., 1975; Julien et al., 2000) and Azotobacteraceae (Socolofsky and Wyss, 1961; Kramer and Socolofsky, 1970). All these spores and spore-like cells share the characteristic of being more resistant to lysis than the vegetative cells and of displaying a reduced metabolic activity. The ability of an organism to produce spores is judged based on morphological and genetic comparisons with these well-studied model organisms forming spores or spore-like cells. However, even though almost all bacteria can enter a dormant state (Rittershaus et al., 2013; Wood et al., 2013), the majority of them do not form spores (asporogenic). Nonetheless, there is evidence of alternative survival structures in asporogenic bacteria (Tanaka et al., 2001; Mulyukin et al., 2003; Suzina et al., 2004, 2006; Lysak and Lapygina, 2018; Filippidou et al., 2019), which are often referred to as “cyst-like” cells as they share some morphological and physiological similarities with cysts (Socolofsky and Wyss, 1961; Sudo and Dworkin, 1973) and show higher resistance than the vegetative cellular state.

Although dormancy and the production of specialized cells is a common strategy in many microorganisms, little information is available about the formation of specialized dormant cells in archaea. Archaea are classified as asporogenic (Moissl-Eichinger et al., 2018) and no evidence of specialized resistant cells has been found so far. Archaea were for a long time thought to be synonyms of extreme environments, since many archaeal isolates were obtained from extreme habitats (Dassarma et al., 2017; Belilla et al., 2019; Medina Chávez et al., 2019). However, this has changed in the last 30 years thanks to the use of culture-dependent and culture-independent techniques, which demonstrated that these microorganisms are also common in non-extreme environments (Francis et al., 2005; Gleeson et al., 2010; Xu et al., 2020). It has been hypothesized that the higher resistance of archaea compared to bacteria originates in the composition of their membrane, which is composed of glycerol-ether lipids that are more chemically resistant than those found in the bacterial membrane (De Rosa et al., 1986; Albers et al., 2000; Konings et al., 2002; Koga and Morii, 2007). More recently, it has been suggested that archaea could also be able to form spores (Fendrihan et al., 2012). Even though there is no direct evidence for spores, the formation of persister cells has been found in *Haloferax volcanii* as a response to stress (Megaw and Gilmore, 2017). Likewise, the induction of dormancy in archaea by viruses has also been observed (Bautista et al., 2015; Gulbudak and Weitz, 2016). Lastly, genes encoding programmed cell death and dormancy in bacteria have been found in archaea (Makarova et al., 2012). Despite all the above-cited evidence, their ability to form specialized resistant cells as a mechanism to survive harsh environmental conditions is not widely recognized. One reason for this could be the inability to recreate environmental conditions that might trigger the formation of specialized resistant cells in archaea. Therefore, investigating specialized resistant cells requires a functional proxy for their formation in the environment.

Dormant microorganisms play an important role in the maintenance of microbial diversity. Their influence in population evolution and microbial processes has been previously highlighted (Jones and Lennon, 2010; Joergensen and Wichern, 2018; Shoemaker and Lennon, 2018). Now that our planet is facing a major ecological challenge in the shape of climate change, the resistant dormant microbial community might represent a seed bank from which new communities can emerge (Jansson and Taş, 2014; Garcia et al., 2020; Wahid et al., 2020). Thus, the ability to assess the lysis-resistant bacterial and archaeal diversity in the environment might help us to better understand the microbial response to environmental change.

The formation of dormant cells has been shown to affect the fitness and ecological distribution of bacteria (Mutlu et al., 2020). Hence, polyextreme environments might be more selective for microorganisms able to produce dormant cells (Filippidou et al., 2016). Polyextreme environments might harbor higher biodiversity of dormant cells than more stable environments where the formation of dormant cells might carry a higher fitness cost. The Salar de Huasco is a high-altitude athalassohaline wetland in the Chilean Altiplano. This salt flat is located at an altitude of 3,800 m.a.s.l. and its salinity ranges from freshwater to saturation (Dorador et al., 2008b). The Salar is known for its polyextreme conditions, with daily temperature ranging from −10 to +25°C, high solar radiation <1,100 W/m^2^, and a negative water balance (Dorador et al., 2010; Cortés-Albayay et al., 2019). All these factors make the Salar de Huasco an environment where microorganisms would benefit from the formation of specialized structures to survive the constantly changing conditions. Furthermore, it has been shown that in this salt flat, there is high biodiversity of endemic bacteria and archaea (Demergasso et al., 2004; Dorador et al., 2008a,b). In this study, we assessed the environmental diversity of lysis-resistant cells in bacteria and archaea by applying a method developed to explore the diversity of endospores in the environment (Wunderlin et al., 2016). This method was extensively validated for its use in bacteria using pure cultures of non-spore-forming Gram-negative and spore-forming Gram-positive bacteria (Wunderlin et al., 2014). To break less-resistant vegetative cells, the method includes chemical and physical lysis prior to DNA extraction of the lysis-resistant fraction. The application of this method enabled the enrichment of endospores and revealed the large biodiversity of other spore formers in environmental samples. In addition, bacterial species that are supposed to be asporogenic were enriched as well (Filippidou et al., 2016; Paul et al., 2018). The unexpected biodiversity observed after the endospore enrichment method, suggests higher biodiversity of highly lysis-resistant bacteria. To assess the microbial diversity of lysis-resistant prokaryotic cells in the Salar de Huasco, we applied the spore separation method (hereafter indicated as “lysis-resistant enrichment”) to samples from sediments and microbial mats.

## Materials and Methods

### Sampling

**FIGURE 1:**
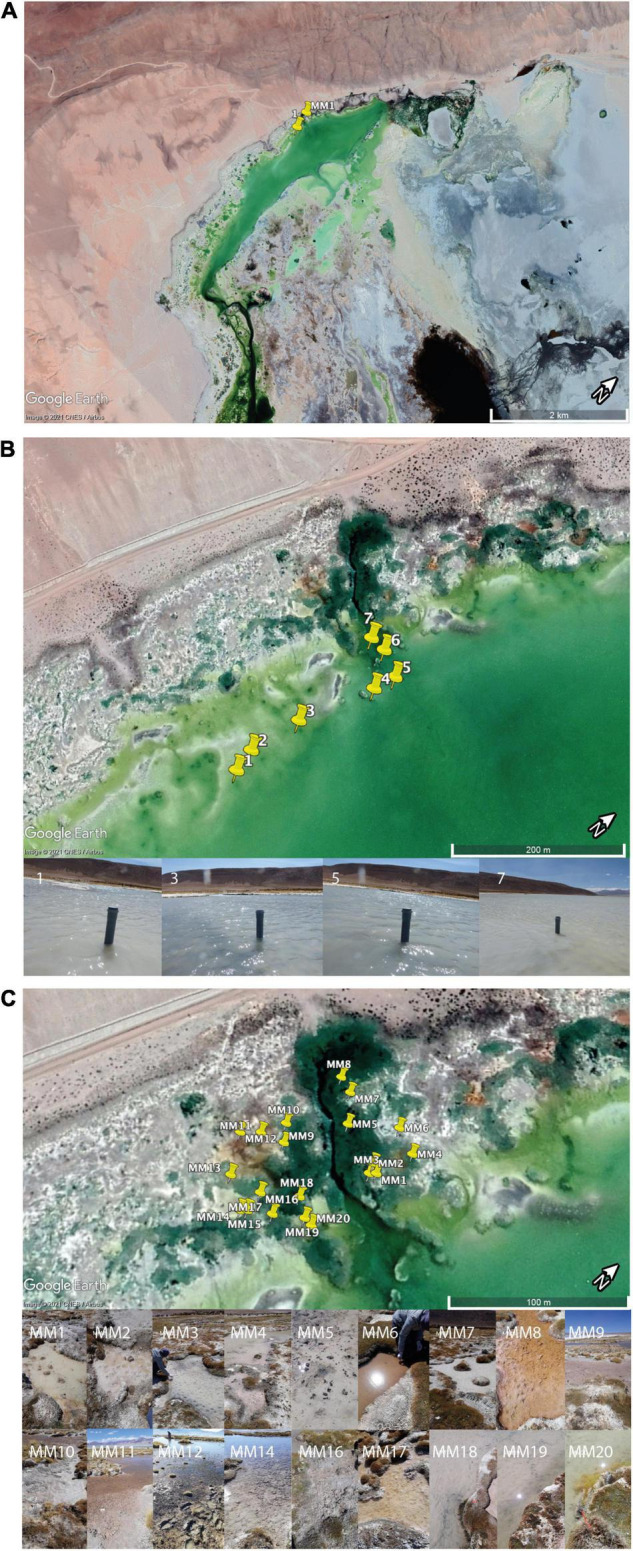
Sampling area. **(A)** An overview of the sampling area indicating the region in which samples for this study were collected. The sampling area was selected based on a previous study (Dorador et al., 2008b) that showed a significant salinity gradient and a large diversity of microbial mats in a limited geographical area. **(B)** Close-up of the area of sampling within the saline lake showing the location of the seven sediment cores. **(C)** Close-up of the area of sampling within ponds containing microbial mats. The images of the 18 ponds are presented to illustrate the diversity of mats studied.

The sampling took place in September 2019. The samples were collected within 24 h from the main saline lake and the small ponds surrounding it. Seven sediment core samples (approximately 7 cm diameter and approximately 4 cm depth) were collected from the main lake, following a saline gradient, with salinity ranging from 53 to 0.706 PSU (Figure 1). The cores were cut on-site to 1 cm subsamples and stored in sterile plastic Petri dishes. Twenty microbial mats samples were collected from eighteen small ponds surrounding the main saline lake. Approximately 40 ml of microbial mats were collected in sterile 50 ml Falcon tubes. At every sampling point salinity, pH, conductivity, water temperature, and dissolved oxygen (%OD) were measured using a Multiparameter Water Quality Meter-HI98194, Hanna Instruments (Woonsocket, Rhode Island, United States), and the coordinates and altitude were also recorded (Supplementary Table 1). During the transport of the samples back to Switzerland, they were stored at room temperature (approximately 20–25°C) for 24 h. After arrival at the lab, the samples were stored at 5°C until processing a week later.

### Biomass Extraction for Indirect DNA Extraction

Attachment to inorganic particles such as sediment can bias the separation of the lysis-resistant fraction by protecting cells from lysis or from enzymatic degradation of DNA (a key step to degrade released DNA from easy to lyse cells during the “lysis-resistant enrichment method”). Therefore, indirect DNA extraction was applied. For each sample, 3 g of material were weighted in ULTRA-TURRAX^®^ disperser tubes (IKA-Werke, Staufen, Germany). Then, 15 ml of Na-Hexametaphosphate (1%) were added and the mixture was homogenized two times at 3,000 rpm for 1 min with the ULTRA-TURRAX^®^ tube disperser workstation system. The sample was left to precipitate for 10 min and the supernatant was collected. This initial step was repeated two times. The two 15 ml supernatants were pooled, centrifuged at 20 × *g* for 1 min to precipitate large particles, and the remaining supernatant was filtrated through a 0.2 μm sterile nitrocellulose filter. For each sample, the filter was cut in half. One half was used to assess the total microbial community by directly extracting DNA and the second half was subjected to the lysis-resistant enrichment method to assess the lysis-resistant fraction. This way the same filter was used to assess both communities (Supplementary Figure 1).

### Lysis-Resistant Enrichment Method and DNA Extraction

To the cellular material on one half of the filter, 900 μl of 1× TE (Tris-EDTA) buffer were added and incubated at 65°C for 10 min shaking at 80 rpm. Afterward, 100 μl of 20 mg/ml lysozyme were added and the sample was incubated at 37°C for 60 min shaking at 80 rpm. Later, 250 μl of 3 N sodium hydroxide (NaOH) and 250 μl of 6% sodium dodecyl sulfate (SDS) solutions were added and the sample was incubated at room temperature for 60 min shaking at 80 rpm. Next, the sample was filtrated in a 0.2 μm sterile nitrocellulose filter, which was then washed two times with 2 ml of sterile physiological water to remove any residue of the solutions used. Once the filter was dry, 450 μl of sterile water, 50 μl of DNase reaction buffer (1×) and 0.5 μl DNase enzyme were added and let stand for 15 min on the filter. The addition of the DNase enzyme and DNase reaction buffer is a vital step to degrade the DNA released by the easy-to-lyse cells prior to the DNA extraction from the lysis-resistant fraction. After 15 min, the solution was filtrated, and once more, the residue was washed with 1 ml of sterile physiological water that was filtrated to wash away the degraded DNA as well as the DNAase solution. Finally, the filters (containing the lysis resistant fraction only) were stored at −20°C until the DNA extraction was performed (Wunderlin et al., 2016). The same DNA extraction method was used in both fractions (Supplementary Figure 1). The FastDNA^®^ SPIN kit for soil (MP Biomedicals, Irvine, CA, United States) was used with a modified protocol that included three successive bead-beating rounds in the first step. The final DNA extracts from the successive bead-beating steps were pooled together (Wunderlin et al., 2013). The DNA was then precipitated with ethanol and resuspended in PCR-grade water. DNA quantification was performed using Qubit^®^ dsDNA HS Assay Kit on a Qubit^®^ 2.0 Fluorometer (Invitrogen, Carlsbad, CA, United States).

### Sequencing and Statistical Analysis

The purified DNA extracts were sent to Fasteris (Geneva, Switzerland) for bacterial and archaeal 16S rDNA amplicon sequencing using an Illumina MiSeq platform (Illumina, San Diego, CA, United States), generating 300 bp paired-end reads. For the bacterial 16S rDNA, the V3–V4 region was amplified using the universal primers Bakt_341F (5′-CCT ACG GGN GGC WGC AG-3′) and Bakt_805R (5′-GAC TACHVG GGT ATCTAA TCC-3′) (Herlemann et al., 2011). For the archaeal 16S rDNA, the V3–V4 region was amplified using the universal primers 340F (5′-CCC TAY GGG GYG CAS CAG-3′) and 806rB (5′-GGA CTA CNV GGG TWT CTA AT-3′) (Bahram et al., 2019). Demultiplexed and trimmed sequence reads provided by Fasteris were processed using QIIME2 (Bolyen et al., 2019) with dada2 (Callahan et al., 2016) for the denoising step. Read lengths were truncated to optimized total nucleotide lengths (based on q-scores), 480 and 484 bases for the 16S and archaea datasets, respectively. These truncated sequences allowed the joining of denoised paired-end reads by at least 12 identical bases to obtain full denoised sequences. Sequences were grouped on amplicon sequence variants (ASVs). The ASVs then obtained were afterward taxonomically classified using QIIME2’s VSEARCH-based consensus taxonomy classifier (Rognes et al., 2016) with the SILVA database (Quast et al., 2013), release 132, for 16S rDNA of bacteria and archaea. The number of ASVs obtained for bacteria was 153,000 and for archaea 2,128. All statistical analyses were performed with RStudio version 1.3.1093 (RStudio Team, 2020), the community and multivariate analyses were performed with the phyloseq (McMurdie and Holmes, 2012) and vegan (Oksanen et al., 2020) packages. The Venn diagram was calculated with the package Venn.Diagram (Chen and Boutros, 2011). The relative abundance was calculated using the Total-Sum Scaling (TSS) normalization. The principal coordinate analysis (PCoA) was calculated based on the weighted UniFrac distances. For the Venn diagram, PCoA, and enrichment analysis, the relative abundance was used. For the statistical test of the alpha diversity and the ANOVA, the raw abundance data were used. The proportion used in the distribution plots was calculated by phylum, the relative abundance of each ASV was divided by the sum of all relative abundance to obtain a proportion, the log base 10 of the proportion was used for the graphical representation. The enrichment proportion was calculated by first adding the relative abundance per genus once in the total fraction and once in the lysis-resistant fraction. The proportion was then calculated by subtracting the lysis-resistant abundance from the total abundance and dividing this by the sum of the lysis-resistant fraction and the total fraction.

## Results

**FIGURE 2:**
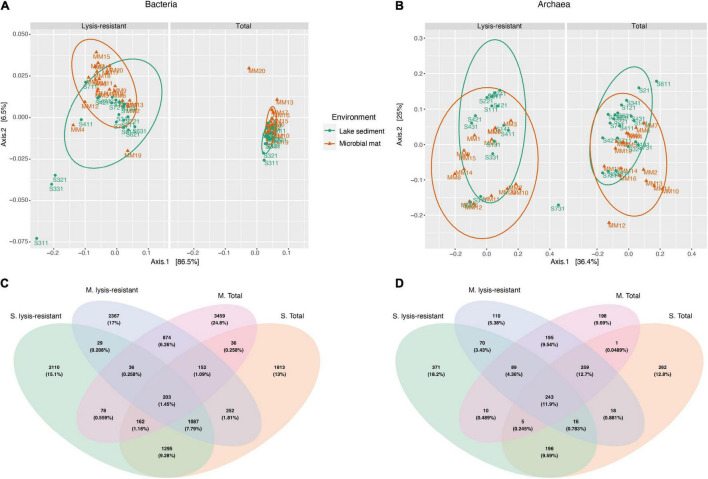
Principal coordinate analysis (PCoA) and Venn diagram of the bacteria and archaea community. **(A)** Weighted UniFrac PCoA of the bacterial community comparing microbial mats (in orange) and lake sediments (in green) in the lysis-resistant (left) and total fractions (right). The ellipses show a 95% confidence level per group. **(B)** PCoA of the archaeal community comparing microbial mats (in orange) and lake sediments (in green) in the lysis-resistant (left) and total fractions (right). The ellipses show a 95% confidence level per group. **(C)** Venn diagram of the bacterial community (top 5,000 of 15,300 ASVs) comparing both environments (M, microbial mats; S, lake sediments) and both community fractions (lysis-resistant and total community). **(D)** Venn diagram of the archaeal community (top 1,000 of 2,128 ASVs) comparing both environments (M, microbial mats; S, lake sediments) and both community fractions (lysis-resistant and total community).

In this study, we analyzed two types of environments: sediment cores from the main saline lake and microbial mats from the small ponds surrounding it. An ordination of the communities using a principal coordinate analysis (PCoA) showed that both the lysis-resistant (i.e., the fraction of the community which persisted after the lysis-resistant enrichment method) and a total fraction (i.e., obtained from the direct DNA extraction) grouped mostly by the type of environment (Figures 2A,B). Although the partial overlap of the communities originating from the two different environments appeared to exist (in particular for the total fraction), a Venn diagram to evaluate the overlap in community composition of both the community type (lysis-resistant and total fraction) and the environment (microbial mat and lake sediment), showed this is not the case. The number of ASVs shared between the same type of environment was higher than between samples from the same community (Figures 2C,D), showing a stronger influence of the type of ecosystem than of the treatment in the overall community composition. This effect was observed for both bacteria and archaea. As the aim of this study was to determine the diversity of lysis-resistant communities, to establish those organisms enriched in this fraction, we compared them to the respective total community of the same environment. Therefore, herein, the two types of environments were analyzed independently, as their biotic characteristics and abiotic conditions were also different.

### Lysis-Resistance in Lake Sediments

Seven sediment core samples were collected along the salinity gradient ranging from 53 to 0.706 PSU in the main saline lake. The pH in the lake ranged between 8.22 and 8.43 (not following a specific gradient). From these samples, sample 5 could not be analyzed as there was not enough sediment to perform the analysis. From the sequencing, we were not able to generate any products for the archaea sample 6 (lysis-resistant only). Although the community analysis was performed in all the samples, for the comparison, only samples that could be paired total vs. lysis-resistant were retained.

#### Bacterial Diversity in Lake Sediments

**FIGURE 3:**
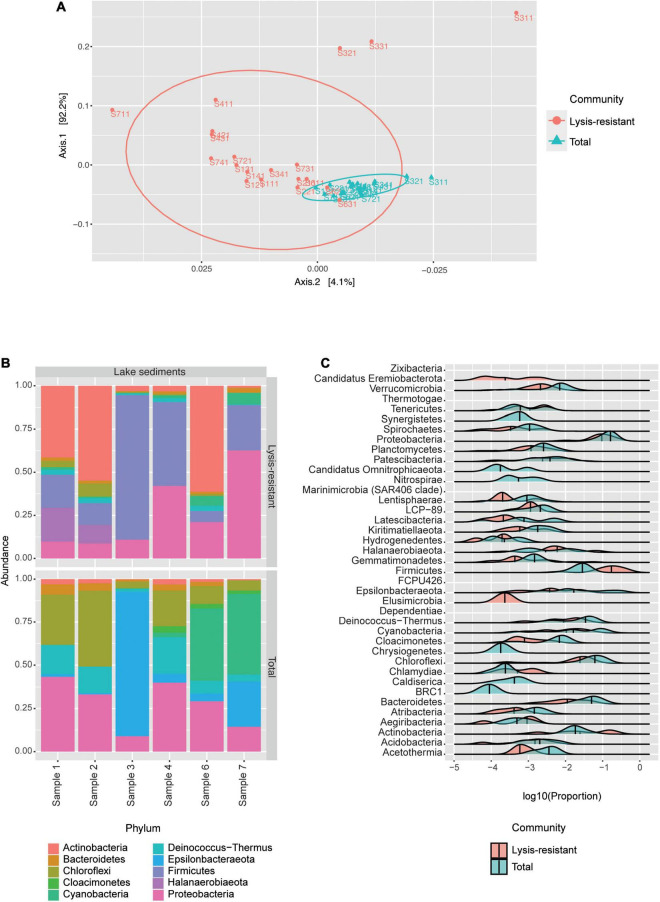
The bacterial community in the lake sediments. **(A)** Weighted UniFrac PCoA of the bacterial community, belonging to the lysis-resistant fraction (red) or the total fraction (blue). The ellipses show a 95% confidence level per group. **(B)** Top 50 bacterial ASVs relative abundance per lake sediment core at the phylum level. **(C)** This plot shows the abundance distribution of the different phyla, for either the lysis-resistant (red) and the total fraction (blue) in the lake sediments, the abundance is shown in log10 of the relative abundance.

The PCoA of the saline lake core samples showed that the bacterial communities were clustered into two groups, the lysis-resistant fraction, and the total community (Figure 3A). In the PCoA the lysis-resistant community samples were more dispersed than the total community. Furthermore, there was no statistical difference (*p*-value = 0.18) between the Shannon alpha diversity of the lysis-resistant and the total fractions (Supplementary Figure 2A). Also, there was no statistically significant correlation between the abiotic parameters (pH, dissolved oxygen, and salinity) and the alpha diversity (data not shown).

We first analyzed the composition of the bacterial community per sediment core by looking at the top 50 ASVs (Figure 3B). The lysis-resistant fraction and the total fraction differed at the phylum level. Both the lysis-resistant and the total fractions included ASVs assigned to Actinobacteria, Bacteroidetes, Chloroflexi, Cyanobacteria, Deinococcus-Thermus, and Proteobacteria, whereas ASVs assigned to Firmicutes and Halanaerobiaeota were only present in the lysis-resistant fraction. In contrast, Cloacimonetes and Epsilonbacteraeota were only present in the total fraction. Moreover, the sediment core from sampling point 3, was significantly different from the other 5 sampling points. The top 50 ASVs in this sample were primarily dominated by Firmicutes in the lysis-resistant fraction and by Epsilonbacteraeota in the total fraction. The enrichment effect of the lysis-resistant method in all detected phyla can also be observed using a distribution plot comparing the relative abundance of given phyla in the two fractions (Figure 3C). As the same filter was used in the assessment of the total and the lysis-resistant community, any cell present in the total fraction capable of withstanding the physical and chemical lysis will show enrichment in the lysis-resistant fraction. When comparing Figures 3B,C, the limitations of only assessing the most abundant ASVs are highlighted. From the 39 different phyla observed in Figure 3C, only 10 were present among the most abundant ASVs, thus overlooking a large part of the bacterial diversity in the lake sediments. The distribution plot also allows the assessment of less abundant phyla and demonstrates the effect that the lysis-resistant method had on them. Figure 3C shows the enrichment of the spore-former phyla Firmicutes and Actinobacteria in the lysis-resistant fraction, as well as the enrichment of non-spore former phyla such as Aegiribacteria, Chlamydiae, Halanaerobiaeota, and Tenericutes in the same fraction. Phyla such as *Candidatus* Eremiobacterota and Elusimicrobia were only present in the lysis-resistant fraction, whereas *Candidatus* Omnitrophicaeota, Nitrospirae, Chrysiogenetes, Caldiserica, and BRC1 only appeared in the total fraction. The phyla Chloroflexi, Proteobacteria, and Bacteroidetes, were among the most abundant phyla in both lysis-resistant and total fractions, and although they were not enriched in the lysis-resistant fraction, they showed a similar distribution in both fractions.

**FIGURE 4:**
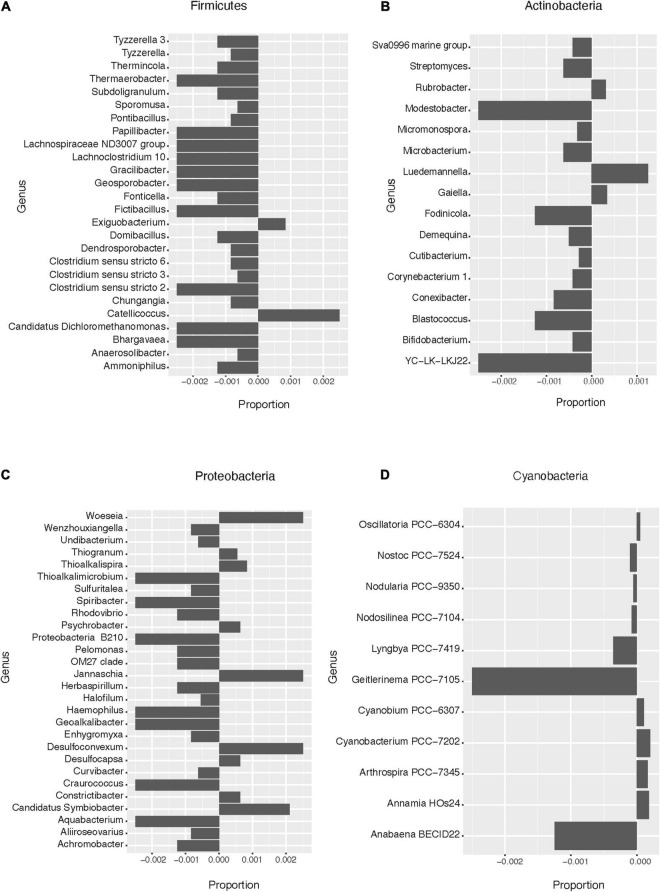
Enrichment of different bacterial genera in the lysis-resistant or total fraction in the lake sediments. Negative values show enrichment in the lysis-resistant fraction, while positive values show enrichment in the total fraction for each genus. **(A)** Firmicutes genus. Proportion threshold at ≤0.0005 and >0.0005. **(B)** Actinobacteria. Proportion threshold at ≤0.00025 and >0.00025. **(C)** Proteobacteria. Proportion threshold at ≤0.0005 and >0.0005. **(D)** Cyanobacteria. Full graph.

Based on the pattern of enrichment described above, representatives of the phyla Firmicutes, Actinobacteria, Proteobacteria, and Cyanobacteria were analyzed in more detail. When assessing the relative abundance of genera within Firmicutes in the total and lysis-resistant fraction (Figure 4A only a subset, full in Supplementary Figure 3), most were clearly enriched in the lysis-resistant fraction. Only 10 genera had a higher abundance in the total fraction. This enrichment in the lysis-resistant fraction was not restricted to genera known to contain spore-forming species, but some asporogenic genera such as *Gracilibacter* and *Chungangia* were also enriched in this fraction (Figure 4A). For Actinobacteria (Figure 4B only a subset, full in Supplementary Figure 4) genera containing known spore former species such as *Streptomyces* and *Micromonospora*, were enriched in the lysis-resistant fraction, but, as in the case of Firmicutes, the lysis-enriched fraction also included non-spore formers genera such as *Nitriliruptor* and *Microbacterium* (Supplementary Figure 4). For both, Proteobacteria and Cyanobacteria, although the mean relative abundance of the lysis-resistant fraction was not higher than the total abundance (Figure 3C), a small fraction of the genera showed a higher abundance in the lysis-resistant fraction (Figures 4C,D; full Figure 4C in Supplementary Figure 5). The enriched fraction included, among others, representatives of the genera *Thioalkalimicrobium*, *Aquabacterium*, and *Spiribacter* in the Proteobacteria and *Nostoc* PCC-7524, *Anabaena* BECID22, and *Geitlerinema* PCC-7105 in Cyanobacteria. A summary of the groups enriched in the bacterial lysis-resistant fraction for other phyla can be found in Supplementary Table 2.

#### Archaeal Diversity in Lake Sediments

**FIGURE 5:**
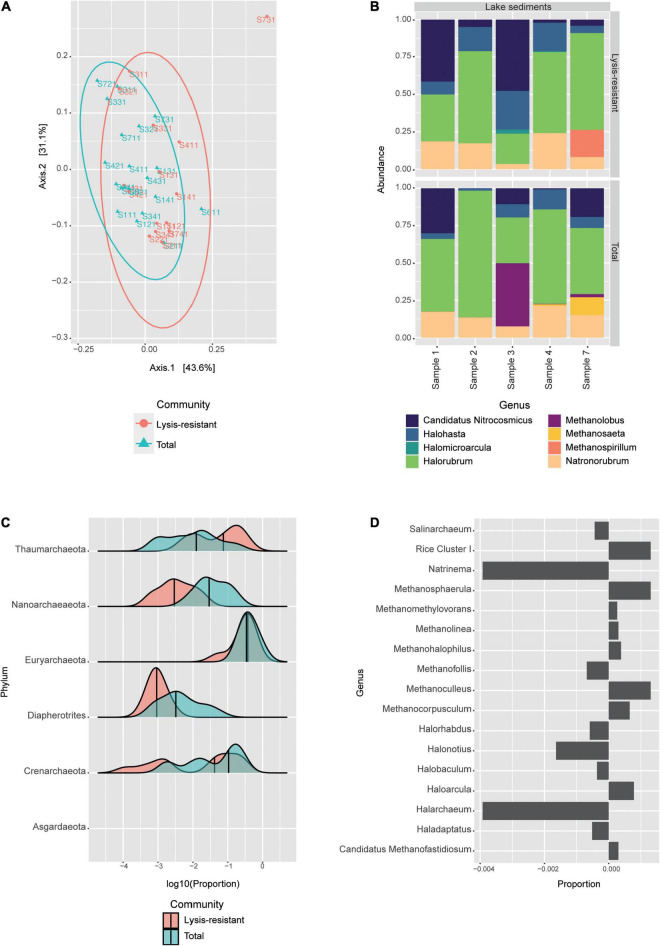
Archaeal community in the lake sediments. **(A)** Weighted UniFrac PCoA of the archaeal community, belonging to the lysis-resistant fraction (red) or the total fraction (blue). The ellipses show a 95% confidence level per group. **(B)** Top 50 archaeal ASVs relative abundance per lake sediment core at the genus level. **(C)** This plot shows the abundance distribution of the different phyla, for either the lysis-resistant (red) and the total fraction (blue) in the lake sediments, the abundance is shown in log10 of the relative abundance. **(D)** Enrichment of Euryarchaeota in the lysis-resistant or total fraction. Negative values show enrichment in the lysis-resistant fraction, while positive values show enrichment in the total fraction. Proportion threshold at ≤0.00025 and >0.00025.

The Shannon alpha diversity of the archaea samples did not show a statistical difference (*p*-value = 0.52) between the samples after the lysis treatment and the samples from the direct DNA extraction (Supplementary Figure 2B). Furthermore, in the PCoA no clear clustering could be observed (Figure 5A). The distribution of the lysis-resistant fraction and the total community overlap in the plot. However, in the sample, each lysis-resistant fraction could be distinguished from the same total community. The communities were largely dominated by two phyla: Euryarchaeota and Thaumarchaeota (data not shown). For archaea, the picture offered by the most abundant ASVs leaves out four phyla, as compared to the phyla observed in the distribution plot, thus underestimating the archaeal diversity. Nonetheless, to further understand the composition of this highly abundant phyla, we went to the genus level for the 50 most abundant ASVs (Figure 5B). The genera that were present both in the total and in the lysis-resistant were *Candidatus* Nitrosocosmicus, *Halohasta*, *Halorubrum*, and *Natronorubrum*, while the genera *Halomicroarcula* (halophilic archaea) and *Methanospirillum* (anaerobic methanogenic) were only present in two and one cores in the lysis-resistant fraction, respectively. In the total community, the presence of *Methanolobus* and *Methanosaeta* was observed.

To assess the enrichment of different archaeal phyla in the lysis-resistant community, we plotted the proportion of each community for each phylum (Figure 5C). The lake sediments sampled from the main Salar showed the presence of 6 phyla: Thaumarchaeota, Nanoarchaeaeota, Euryarchaeota, Diapherotrites, Crenarchaeota, and in lower abundance Asgardaeota. From these phyla, only Thaumarchaeota showed enrichment in the lysis-resistant fraction, while Euryarchaeota had similar means in both fractions. Moreover, it can also be observed that Thaumarchaeota and Crenarchaeota showed more than one distribution in the data set, indicating that there was also a set of less abundant ASVs with high distribution in both types of community fractions. For Thaumarchaeota, there were two enriched genera in the lysis-resistant fraction, these were *Nitrososphaera* and *Candidatus* Nitrosocosmicus, while unidentified genera were enriched in the total fraction (data not shown). Both *Nitrososphaera* and *Candidatus* Nitrosocosmicus are soil ammonia oxidizers from which no resistant cell form is known. Although the Euryarchaeota phyla did not show significant enrichment in the lysis-resistant fraction (Figure 5D, only a subset full in Supplementary Figure 6), we observed that many genera were enriched in the lysis-resistant fraction (Figure 5D). Those included *Natrinema*, *Halonotius*, and *Halarchaeum*. These genera belong to the Halobacteria class, known to be halophilic archaea found mainly in water-saturated or nearly saturated with salt, such as the Salar de Huasco.

### Lysis-Resistance in Microbial Mats

Twenty samples of microbial mats were collected from 18 small ponds surrounding the main saline lake. The samples varied in color, texture, and location. The pH in these ponds ranged between 7.67 and 9.74, and the salinity from 0.41 to 4.22 PSU. Sequencing data were collected for all the samples for bacteria. However, for archaea, we were not able to generate sequencing products for all the samples. The samples missing data were samples 12, 15, 16, and 17 (in the lysis-resistant fraction), and samples 8, 15, and 18 (total fraction). Although the community analysis was performed in all the samples, for the comparison only samples that could be paired, total vs. lysis-resistant were retained.

#### Bacterial Diversity in Microbial Mats

**FIGURE 6:**
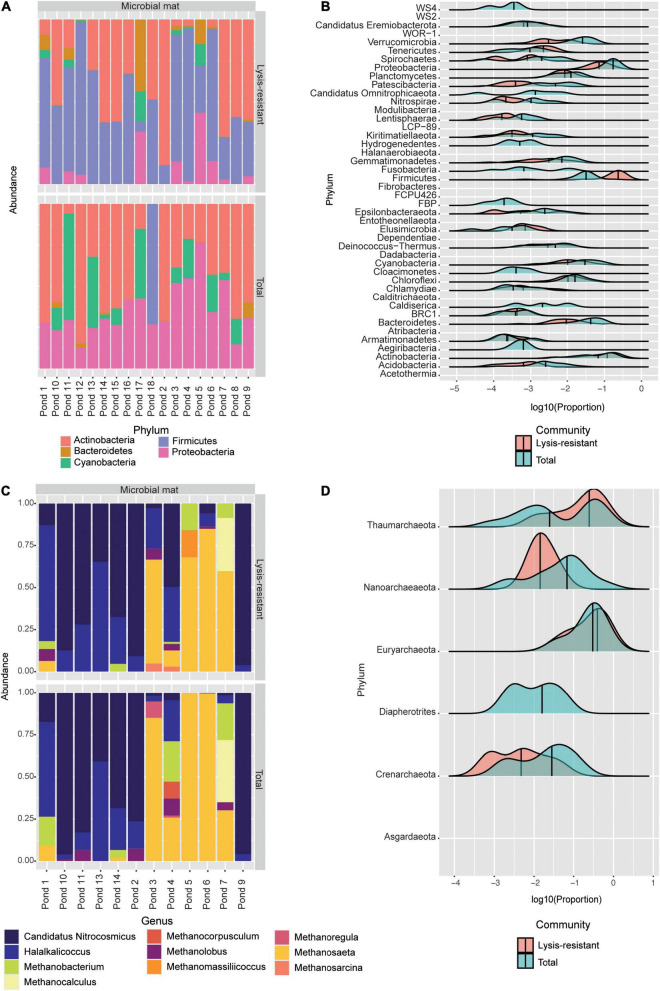
**(A)** Top 50 bacterial ASVs relative abundance per pond at the phylum level. **(B)** This plot shows the abundance distribution of the different bacterial phyla, for either the lysis-resistant (red) or the total fraction (blue) in the microbial mats. The abundance is shown in log10 of the relative abundance. **(C)** Top 50 archaeal ASVs relative abundance per pond at the genus level. **(D)** This plot shows the abundance distribution of the different archaeal genus, for either the lysis-resistant (red) or the total fraction (blue) in the microbial mats. The abundance is shown in log10 of the relative abundance.

The PCoA of the microbial mat samples showed the same clustering as the one observed in the lake sediments (Supplementary Figure 7A). The total community was tightly clustered except for one sample, MM20, which was clustered with the lysis-resistant fraction. On the other hand, the lysis-resistant fraction had a broader distribution. The difference in alpha diversity between the two fractions was significant in the case of bacteria (Supplementary Figure 7B). There was no significant statistical correlation between the abiotic parameters and alpha diversity. The most abundant ASVs in the microbial mat and the lake sediments belonged to the same five main phyla: Actinobacteria, Bacteroidetes, Cyanobacteria, Firmicutes, and Proteobacteria (Figure 6A). However, the abundance of these phyla is significantly different between the two communities (lysis-resistant and total). As it can be observed, in the microbial mats, the total fraction was mainly composed of Actinobacteria, Proteobacteria, and Cyanobacteria, while Firmicutes only appeared in two of the sampling points (pond 4 and pond 18). However, in the lysis-resistant fraction, the most abundant phyla were Firmicutes and Actinobacteria; Proteobacteria were present in almost all samples but in lower relative abundance and Cyanobacteria were present in only seven ponds.

**FIGURE 7:**
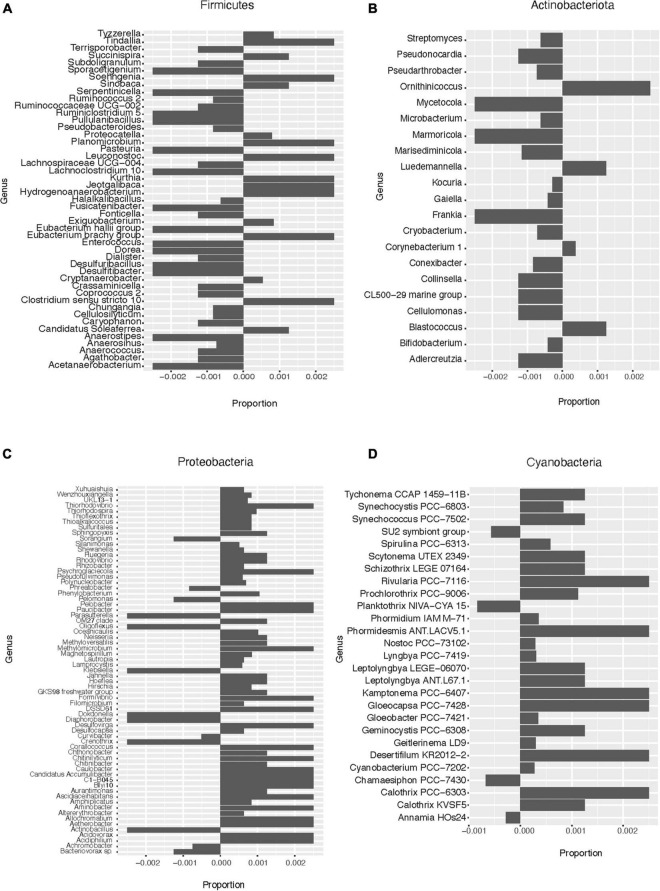
Enrichment of different bacterial genera in the lysis-resistant or total fraction in the microbial mats. Negative values show enrichment in the lysis-resistant fraction, while positive values show enrichment in the total fraction for each genus. **(A)** Firmicutes genus. Proportion threshold at ≤0.0005 and >0.0005. **(B)** Actinobacteria. Proportion threshold at ≤0.00025 and >0.00025. **(C)** Proteobacteria. Proportion threshold at ≤0.0005 and >0.0005. **(D)** Cyanobacteria. Proportion threshold at ≤0.00025 and >0.00025.

To obtain a general idea of the bacterial diversity and the enrichment of the different phyla in the microbial mat community, we plotted the proportion of each community for each phylum (Figure 6B). Here, 44 phyla were identified while in the lake sediments only 39 phyla were identified. Furthermore, these two environments shared only 1.45% of their bacterial ASVs (Figure 2C). When comparing the enrichment of specific phyla in the lysis-resistant community in microbial mats (Figure 6B) vs. lake sediments (Figure 3C), Firmicutes, Proteobacteria, Actinobacteria, Cyanobacteria, Chlamydiae, Chloroflexi, *Candidatus* Omnitrophicaeota, and Caldiserica showed the same distribution. On the other hand, the phyla *Candidatus* Eremiobacterota, and Elusimicrobia were only present in the lysis-resistant fraction. Nitrospirae and BRC1 were only present in the total fraction in the lake sediments, while in the microbial mat they are present in both fractions. The opposite was true for the phyla Hydrogenedentes, Cloacimonetes, and Aegiribacteria, which were only present in the total fraction in the microbial mats but were present in both fractions in the lake sediments. A more detailed assessment of the enriched phyla of the microbial mats is shown in Figure 7 (only a subset, full in Supplementary Figures 8–11). The majority of the genus in Firmicutes (Figure 7A) showed a higher abundance in the lysis-resistant fraction as compared to the total fraction. *Clostridium*, *Bacillus*, *Paenibacillus*, and *Sporosarcina* are known spore formers found among those genera enriched. In addition, genera not known to form spores but with higher abundance in the lysis-resistant fraction, such as *Enterococcus* and *Dorea*, were also detected. In the case of Actinobacteria (Figure 7B), only six of 36 genera present showed higher abundance in the total fraction in comparison to the lysis-resistant fraction (Supplementary Figure 9). Not only known spore formers such as *Streptomyces*, *Micromonospora*, and *Frankia* showed higher abundance in the lysis-resistant fraction, but also asporogenic genera such as *Mycetocola*, *Conexibacter*, and *Adlercreutzia*. In the other two phyla with known spore formers, Proteobacteria, and Cyanobacteria, most of the genera had higher abundance in the total fraction than in the lysis-resistant fraction (Figures 7C,D). The Cyanobacteria genera enriched in the lysis-resistant fraction were SU2 symbiont group, *Planktothrix* NIVA-CYA-15, *Nostoc* PCC7524, *Chamaesiphon* PCC7430, and *Annamia* HoS24, from which only *Nostoc* PCC7524 is known to produce akinetes. For Proteobacteria the most enriched genera in the lysis-resistant fraction were *Parasutterella*, *Oligoflexus*, *Klebsiella*, *Dokdonella*, and *Actinobacillus*, all of which are not known to form spores.

**FIGURE 8:**
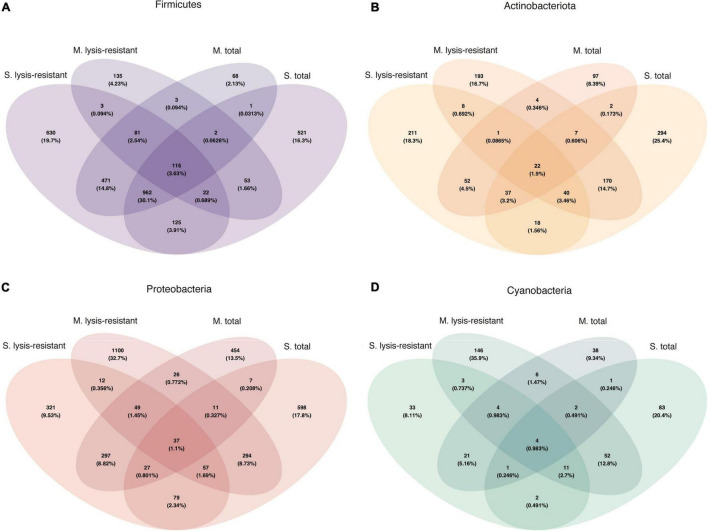
Venn diagram of the bacterial community for each fraction and environment. **(A)** Firmicutes in the 5,000 top ASVs. **(B)** Actinobacteria in the 5,000 top ASVs. **(C)** Proteobacteria in the 5,000 top ASVs. **(D)** Cyanobacteria in the 5,000 top ASVs.

A comparison of the genera of Firmicutes, Actinobacteria, Proteobacteria, and Cyanobacteria shared between the microbial mats and lake sediments showed that only 3.63, 1.9, 1.1, and 0.98% ASVs were shared, respectively (Figures 8A–D). When looking at the shared ASVs per fraction (lysis-resistant and total) the maximum number shared is 0.737%, for Cyanobacteria in the lysis-resistant fraction, and 0.246% in the total fraction. This confirms that although most of the enriched phyla were shared between the two environments, the ASVs from those phyla were distinct.

#### Archaeal Diversity in Microbial Mats

As observed in the lake sediments, the archaeal PCoA did not show any defined clustering either for the lysis-resistant or for the total community (Supplementary Figure 7C). Also, there was no statistical difference in the alpha diversity of these two communities (Supplementary Figure 7D). At the phylum level, only Euryarchaeota and Thaumarchaeota were present. The community composition of the top 50 ASVs was assessed per sampled pond at the genus level (Figure 6C). Most genera could be found in both the lysis-resistant and the total fraction. On the other hand, the genus *Methanocorpusculum* was only present in the total fraction and the genera *Methanomassiliicoccus* and *Methanosarcina* were only present in the lysis-resistant fraction. Additionally, these genera were only present in a few samples. The genera *Methanocorpusculum* and *Methanomassiliicoccus* were only present in one pond each (pond 4 and pond 5, respectively). The two microbial mats collected from pond 4 were highly structured, pink submerged mats with a salinity of 0.738 PSU and a pH of 7.89, while the sample from pond 5 was a brown submerged mat presenting the same salinity and pH as pond 4. The genus *Methanosarcina* was only found in ponds 3 (black-green submerged mat, salinity of 0.412 PSU, and pH of 7.9) and 4. Meanwhile, the top 50 ASVs of ponds 5 and 6 (whitish submerged mat, salinity of 0.738 PSU, and pH of 7.89) were dominated by the genus *Methanosaeta* in the total fraction, whereas the lysis-resistant fraction of the same ponds also presented the genera, *Candidatus* Nitrosocosmicus, *Halalkalicoccus*, *Methanobacterium*, and *Methanocalculus*. A larger archaeal diversity was revealed when assessing the enrichment of each phylum (Figure 6D). Here it can be observed that as for the lake sediments, only the Thaumarchaeota was enriched in the lysis-resistant fraction. All other phyla were enriched in the total fraction, while Diapherotrites were only present in the total fraction.

**FIGURE 9:**
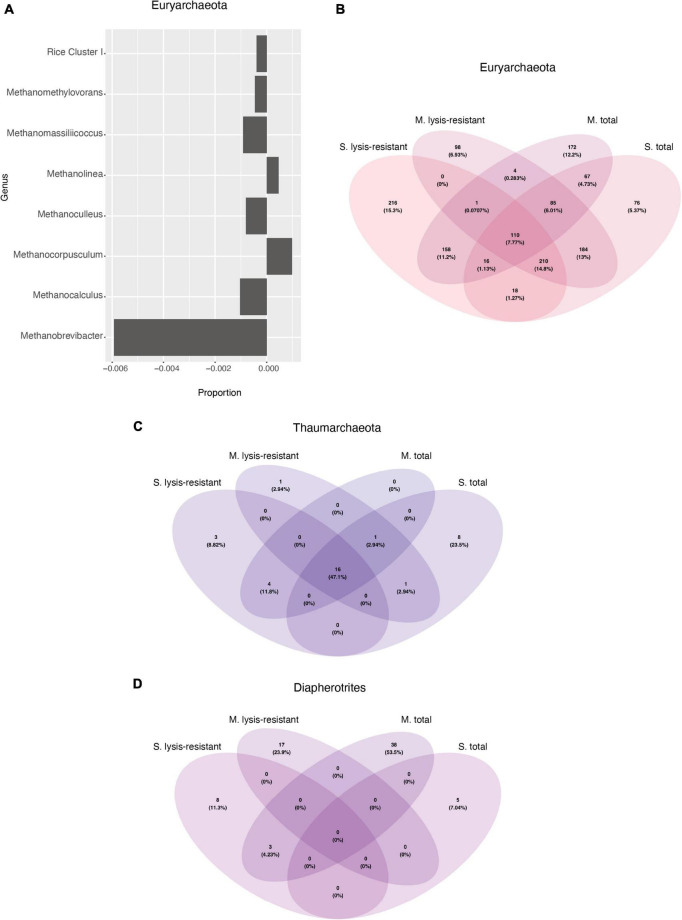
**(A)** Enrichment of different Euryarchaeota genus in the lysis-resistant or total fraction in the microbial mats. Negative values show enrichment in the lysis-resistant fraction, while positive values show enrichment in the total fraction for each genus. Threshold at ≤0.00025 and >0.00025. **(B)** Venn diagram of the Euryarchaeota in the 5,000 top ASVs for each fraction and environment. **(C)** Venn diagram of the Thaumarchaeota in the 5,000 top ASVs for each fraction and environment. **(D)** Venn diagram of the Diapherotrites in the 5,000 top ASVs for each fraction and environment.

A closer inspection of the enriched phyla showed that in the Thaumarchaeota phylum there were only two genera identified *Candidatus* Nitrosocosmicus and Nitrososphaera from which only the latter showed enrichment in the lysis-resistant fraction (data not shown). In the case of the Euryarchaeota phylum, many genera showed no enrichment in either fraction, but the same relative abundance in both (Figure 9A, only a subset, full in Supplementary Figure 12). *Methanobrevibacter*, *Mathanocalculus*, *Methanoculleus*, and *Methanomassiliicoccus* were genera enriched in the lysis-resistant fraction, with *Methanobrevibacter* showing the highest enrichment in this fraction. These four genera are all known to contain methanogenic archaea, and the formation of resistant modified structures is not yet known. In the case of the main genera enriched in the total fraction, *Methanocorpusculum*, *Methanolinea*, and *Candidatus* Methanofastidiosum, all three are methanogenic anaerobic archaea. Additionally, the Venn diagram of Euryarchaeota (Figure 9B), showed that the total fractions of the microbial mats and the lake sediments share more ASVs (4.73%) than each of the fractions from the same environment (lake sediment 1.25% and microbial mat 0.28% ASVs). In the case of Thaumarchaeota and Diapherotrites, the diversity in each environment and fraction appears to be unique (Figures 9C,D).

## Discussion

The overall diversity of the bacterial and archaeal communities in the two types of samples analyzed in the Salar de Huasco agrees with previous studies in this site and similar environments. The high relative abundance of Proteobacteria and Actinobacteria in both the lake sediments and microbial mats (lysis-resistant and total fraction) is in agreement with previous studies assessing the bacterial diversity in the site (Aguilar et al., 2016; Molina et al., 2018a; Castro-Severyn et al., 2021), as well as in other saline lakes around the world (Qin et al., 2019; Mehta et al., 2021). Furthermore, the high relative abundance of Chloroflexi in the lake sediments and of Cyanobacteria in the microbial mats is also in agreement with previous studies (Dorador et al., 2008b; Rasuk et al., 2016; Prieto-Barajas et al., 2018; Castro-Severyn et al., 2021). Moreover, although the most abundant phyla (Proteobacteria, Cyanobacteria, Actinobacteria, and Bacteroidetes) seem to be present in both environments (lake sediments and microbial mats), the number of shared ASVs between them was low. Such differences in the community composition of each environment have been previously observed at different sampling stations in Salar de Huasco (Dorador et al., 2020). Thaumarchaeota and Euryarchaeota were the dominant archaea phyla in the Salar de Huasco. These phyla have been previously reported to be highly abundant in sediments and water (Dorador et al., 2010; Molina et al., 2018b). On the other hand, our results contrast to those found by Molina et al. (2018b), where they found that the archaeal phylum *Candidatus* Parvarchaeota was the most abundant in both microbial mats and sediments, whereas in our community analysis this phylum was not found. Genera containing methanogenic archaea such as *Methanobacterium*, *Methanocorpusculum*, *Methanoregula*, *Methanosaeta*, and *Methanospirillum*, were among the most abundant in the microbial mats as reported in previous studies (Dorador et al., 2010; Molina et al., 2018b). On the other hand, halophilic archaea, such as *Halalkalicoccus*, *Halohasta*, *Halomicroarcula*, and *Halorubrum*, were abundant in lake sediments, something that was observed previously in samplings held during the Austral winter season as in our case (Dorador et al., 2013). A high number of uncultured and/or unidentified ASVs belonging to both bacteria and archaea was also detected. This is not only characteristic of the Salar de Huasco but many extreme environments (Bull et al., 2016; Dorador et al., 2020; Pascoal et al., 2021).

This study used, for the first time, an experimental approach developed originally for the enrichment of endospores to evaluate the diversity of potentially dormant microorganisms in this polyextreme environment. The method used here was initially validated by testing the survival of endospore-formers and non-endospore formers to chemical and physical lysis. The final treatment was shown to destroy vegetative cells, but not highly resistant endospores, which are only found in Firmicutes. In addition to the enrichment of Firmicutes, other taxa were also detected and therefore the enriched fraction was considered to contain diverse lysis-resistant cells as, for many of the enriched taxa, endospore formation (or other forms of sporulation) was unproven (Wunderlin et al., 2014). The results presented here for the Salar de Huasco confirmed that lysis-resistance is a specialized mechanism found only in a fraction of the bacterial population, resulting in the enrichment of phyla in which species are known to form spores, such as Firmicutes and Actinobacteria. The high abundance of Firmicutes and Actinobacteria in the lysis-resistant fraction is in agreement with Kearney et al. (2018), who also found these two phyla highly enriched in human fecal samples after the application of a modified version of the same method. Furthermore, not only known spore formers were enriched in the lysis-resistant fraction, but also genus not yet known to form resistant specialized structures were enriched in this fraction. This includes, for instance, *Gracilibacter* (Lee et al., 2006) and *Enterococcus* (Schleifer and Kilpper-Balz, 1984; Byappanahalli et al., 2012) in Firmicutes, and *Nitriliruptor* (Sorokin et al., 2017) and *Microbacterium* (Gneiding et al., 2008; Fidalgo et al., 2016) in Actinobacteria. Their enrichment in the lysis-resistant fraction, suggests that a highly resistant cell form might be responsible for enhancing their survival in the environment.

In Proteobacteria, the formation of resistant specialized structures is known in the *Azotobacter* genus (Høidal et al., 2000; Rejman and Kozubek, 2004) and in the *Myxococcales* order (Kottel et al., 1975; Julien et al., 2000; Garcia et al., 2009; Huntley et al., 2011; Mohr et al., 2012). A member of the latter, the genus *Sorangium*, was enriched in the spore fraction of the microbial mats, while a second one (*Phaselicystis*) was enriched in the lake sediment samples. The formation of exospores (a type of spore usually produced by members of the Actinobacteria phyla) has been described in *Thermosporothrix hazakensis* (Yabe et al., 2010a,b) and *Thermogemmatispora onikobensis* (Yabe et al., 2011), two species within the class Ktedonobacter in Chloroflexi. Although these two genera were not present in our results, the enrichment of *Nitrolancea* (Sorokin et al., 2012), *Candidatus* Chloroploca (Bryantseva et al., 2021), and *Chloronema* (Chiriac et al., 2017) in the lysis-resistant fraction, could indicate a larger distribution of exospore-like cells within Chloroflexi.

The enrichment in the lysis-resistant fraction of other taxa that are composed so far of exclusively culturable asporogenic species such as Tenericutes, Chlamydiae, Aegiribacteria, Halanaerobiaeota, *Candidatus* Eremiobacterota (formerly known as WPS-2), and Elusimicrobia is noteworthy. Although for none of them spore-like cells are known, the extent to which culturable type strains are a good representation of their environmental counterparts is still unclear. It has been shown that the “domestication” of bacterial isolates can affect their metabolism, morphology, fitness, sporulation, pathogenesis, and resistance (Fux et al., 2005; Sastalla and Leppla, 2012; Eydallin et al., 2014; Norris et al., 2020). In the case of representatives within Tenericutes and Chlamydiae, their lifestyle might provide a mechanism to explain their enrichment in the lysis-resistant fraction. In the case of Tenericutes, *Mollicutes* is the only class described to date. Members of this class, are characterized by the lack of a cell wall and by the establishment of close association with animal and plant hosts (Skennerton et al., 2016). Likewise, some species within the phylum Chlamydiae, although also asporogenic, are known for the production of elementary bodies, which are non-replicating infectious particles. The elementary bodies are released from infected cells and can survive in the environment until they encounter a new host (Elwell et al., 2016). Thus, the fact that Tenericutes and Chlamydiae are obligated intracellular pathogens might explain their enrichment in the lysis-resistant fraction, as it would be necessary to first break the host cell to attain their DNA. In addition, the enrichment of Chlamydiae has been previously observed after the application of the lysis-resistant method in sediments from the Rhone delta in Lake Geneva (Madueño et al., 2018) and in permafrost from Fairbanks, Alaska (Burkert et al., 2019).

In comparison to bacteria, in archaea, lysis resistance seems to be widely spread as there was no clear grouping of the lysis-resistant fraction relative to the total fraction in the PCoA. Also, there was no statistical difference in the alpha diversity between these two communities. The widespread enrichment of archaeal ASVs in the lysis-resistant fraction might be due to the high tolerance of archaea to lysis. The assessment of lysis resistance of archaeal cells faces a few limitations. One of them is the large fraction of unassigned ASVs found in our data set. Another one is that while the method to enrich lysis-resistant cells was extensively validated to enrich lysis-resistant bacterial cells, until now it has not been validated for archaea, and thus the results obtained in the case of archaea should be interpreted with caution. Nevertheless, the same filter was used for the assessment of the bacterial and archaeal community, and it is unlikely that archaeal DNA released during the physical and chemical lysis has a higher resistance to the DNase digestion than the bacterial DNA (Miettinen et al., 2019; Sorensen et al., 2021). Moreover, although some genes encoding programmed cell death and dormancy in bacteria have been found in archaea (Makarova et al., 2012), the lack of markers such as the regulator protein Spo0A (Molle et al., 2003), and calcium dipicolinic acid (Ca-DPA) (Fichtel et al., 2007) in Firmicutes or the protein SsgA in *Streptomyces* (Van Wezel et al., 2000), makes the identification and characterization of specialized resistant cells in archaea more difficult. Even though all these limitations make the assessment of the archaeal results challenging, the enrichment of two genera, *Natrinema* and *Halarchaeum*, is worth mentioning. Interestingly, cyst-like cells have been produced for *Natrinema pallidum* (Suzina et al., 2006). Also, cells belonging to the genus *Halarchaeum* have been isolated from 100 to up to 300 million years old salt deposits (Gruber et al., 2004; Schubert et al., 2010). These two genera were the most enriched of Euryarchaeota detected in the lysis-resistant fraction of the lake sediments, suggesting that the enrichment might reflect the existence of cyst-like or other types of resistant archaeal cells in the environment.

In summary, the application of the lysis-resistant method in the samples collected at the Salar de Huasco revealed a high diversity of lysis-resistant bacteria and potentially in archaea. Some bacteria are known to form resistant specialized structures, but the results shown here suggest a broader diversity of lysis-resistant bacteria. The culture-independent method used in this study also shows the presence of archaea that display similar resistance to lysis as lysis-resistant bacteria, based on their enrichment in the lysis-resistant fraction. Moreover, to our knowledge, this is the first study investigating lysis-resistance diversity in environmental archaea. To better understand both bacterial and archaeal production of lysis-resistant cells in the environment, new methods that enable the identification of lysis-resistant cells without the limitations of culturing and that also allow further analysis of the cells (e.g., microscopy, resistant tests) are still needed. Furthermore, in the face of a future in which the planet has an even more unpredictable and changing climate, understanding the diversity of microorganisms with the ability to survive highly fluctuating environmental conditions might help us predict the response of microbial communities to climate change.

## Data Availability Statement

The datasets presented in this study can be found in online repositories. The names of the repository/repositories and accession number(s) can be found below: NCBI GenBank – PRJNA786740.

## Author Contributions

AC, PJ, GC, MF, and CD made major contributions to the acquisition of the data. AC and PJ made major contributions to the analysis or interpretation of the data. All authors proofread and approved the manuscript.

## Conflict of Interest

The authors declare that the research was conducted in the absence of any commercial or financial relationships that could be construed as a potential conflict of interest.

## Publisher’s Note

All claims expressed in this article are solely those of the authors and do not necessarily represent those of their affiliated organizations, or those of the publisher, the editors and the reviewers. Any product that may be evaluated in this article, or claim that may be made by its manufacturer, is not guaranteed or endorsed by the publisher.
