# Supplementary material for: Diversity of Lysis-Resistant Bacteria and Archaea in the Polyextreme Environment of Salar de Huasco
Source: Front Microbiol. 2022 Apr 25;13:826117. doi: 10.3389/fmicb.2022.826117 (PMC9847572; doi:10.3389/fmicb.2022.826117)
Supplement: Supplementary file 1 [file Data_Sheet_1.PDF]

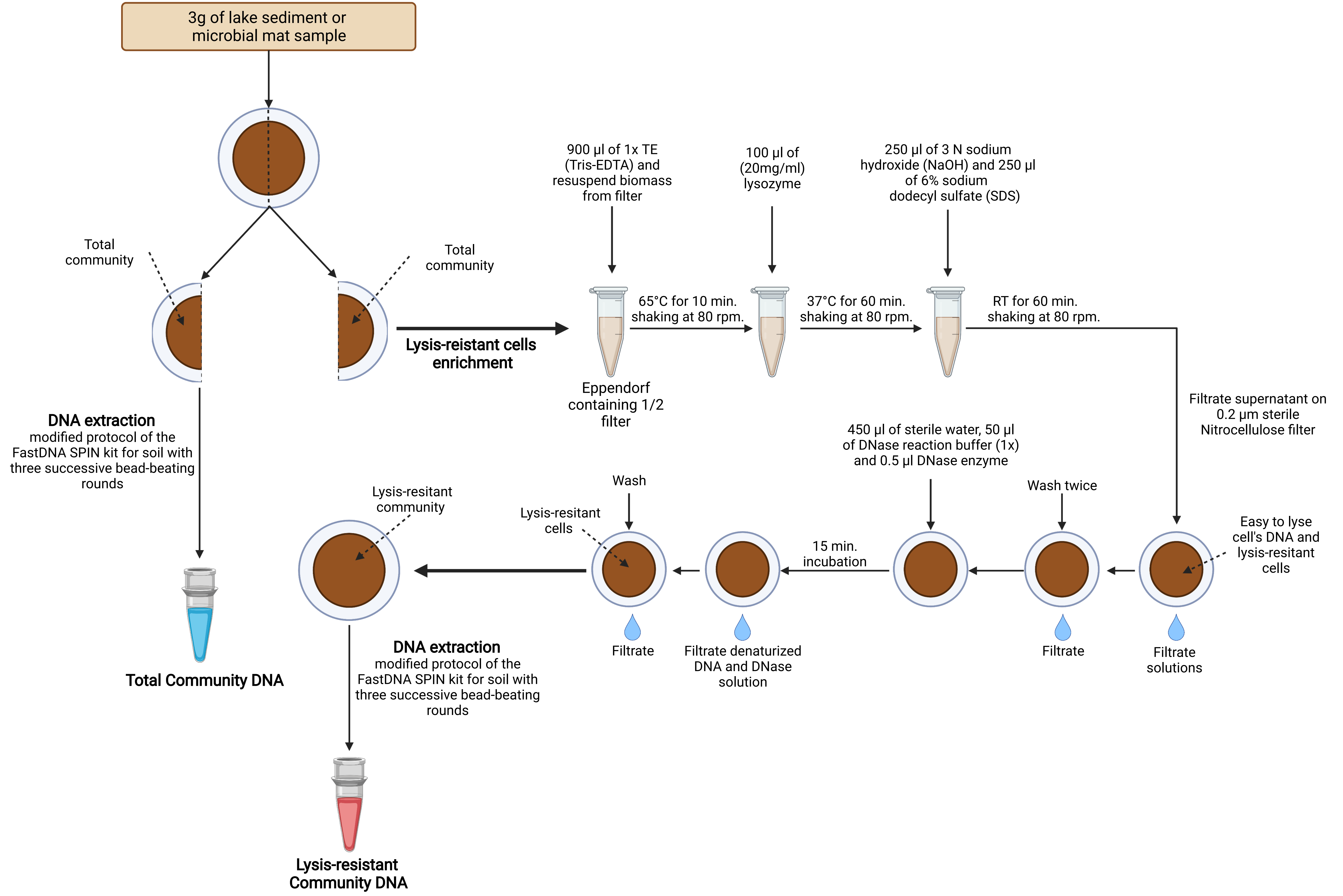

Supplementary Figure 1 . Overview of the method used to obtain the DNA from the total and the lysis-resistant community from lake sediment and microbial mat samples. Created with BioRender.com
