# Supplementary material for: Diversity of Lysis-Resistant Bacteria and Archaea in the Polyextreme Environment of Salar de Huasco
Source: Front Microbiol. 2022 Apr 25;13:826117. doi: 10.3389/fmicb.2022.826117 (PMC9847572; doi:10.3389/fmicb.2022.826117)
Supplement: Supplementary file 2 [file Data_Sheet_2.PDF]

Lake sediments

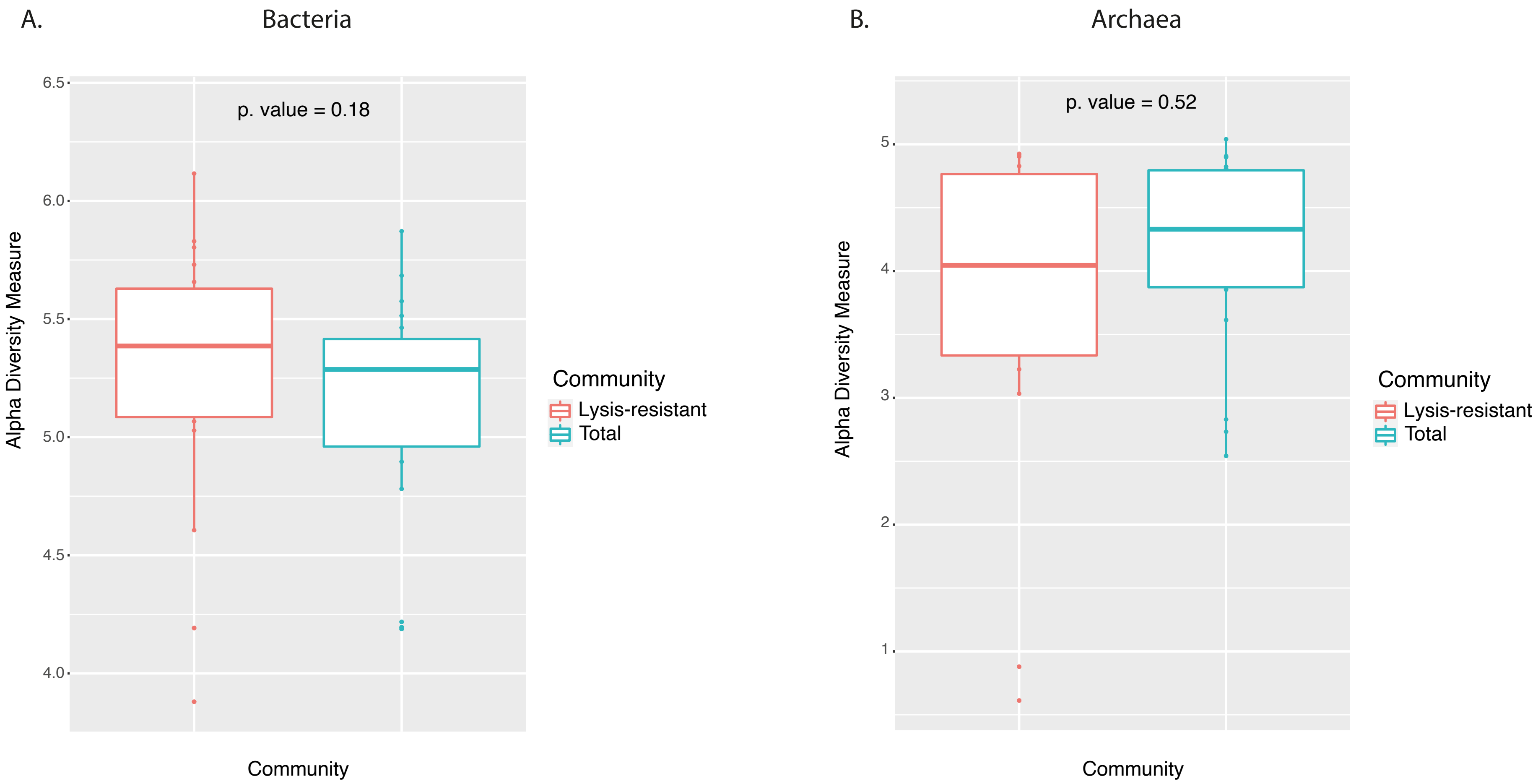

Supplementary Figure 2. Alpha diversity of the lake sediments **A**. Bacteria. **B.** Archaea
