# Supplementary material for: Diversity of Lysis-Resistant Bacteria and Archaea in the Polyextreme Environment of Salar de Huasco
Source: Front Microbiol. 2022 Apr 25;13:826117. doi: 10.3389/fmicb.2022.826117 (PMC9847572; doi:10.3389/fmicb.2022.826117)
Supplement: Supplementary file 5 [file Data_Sheet_5.PDF]

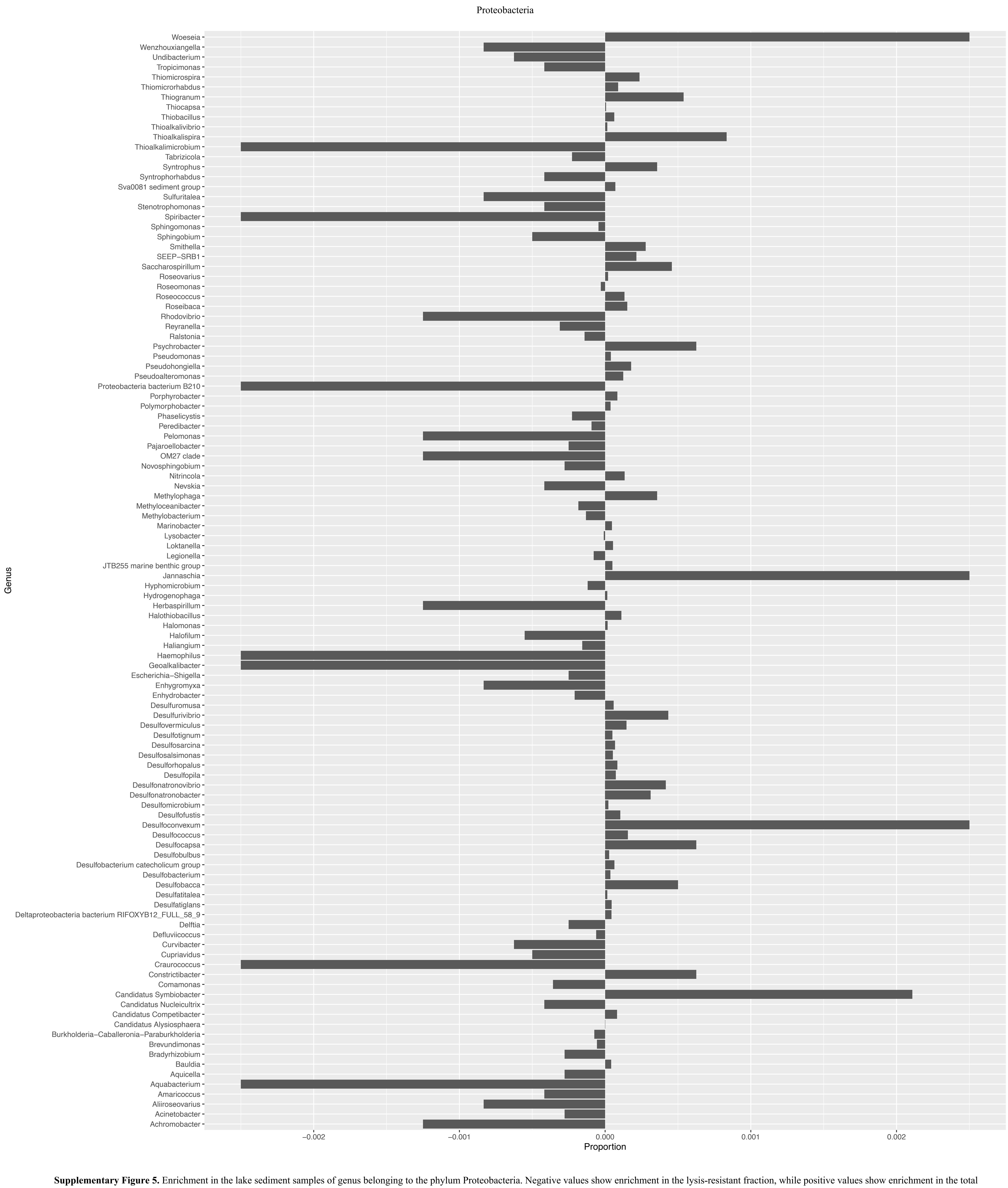

Supplementary Figure 5. Enrichment in the lake sediment samples of genus belonging to the phylum Proteobacteria. Negative values show enrichment in the lysis-resistant fraction, while positive values show enrichment in the total fraction for each genus.
