# Supplementary material for: Diversity of Lysis-Resistant Bacteria and Archaea in the Polyextreme Environment of Salar de Huasco
Source: Front Microbiol. 2022 Apr 25;13:826117. doi: 10.3389/fmicb.2022.826117 (PMC9847572; doi:10.3389/fmicb.2022.826117)
Supplement: Supplementary file 7 [file Data_Sheet_7.PDF]

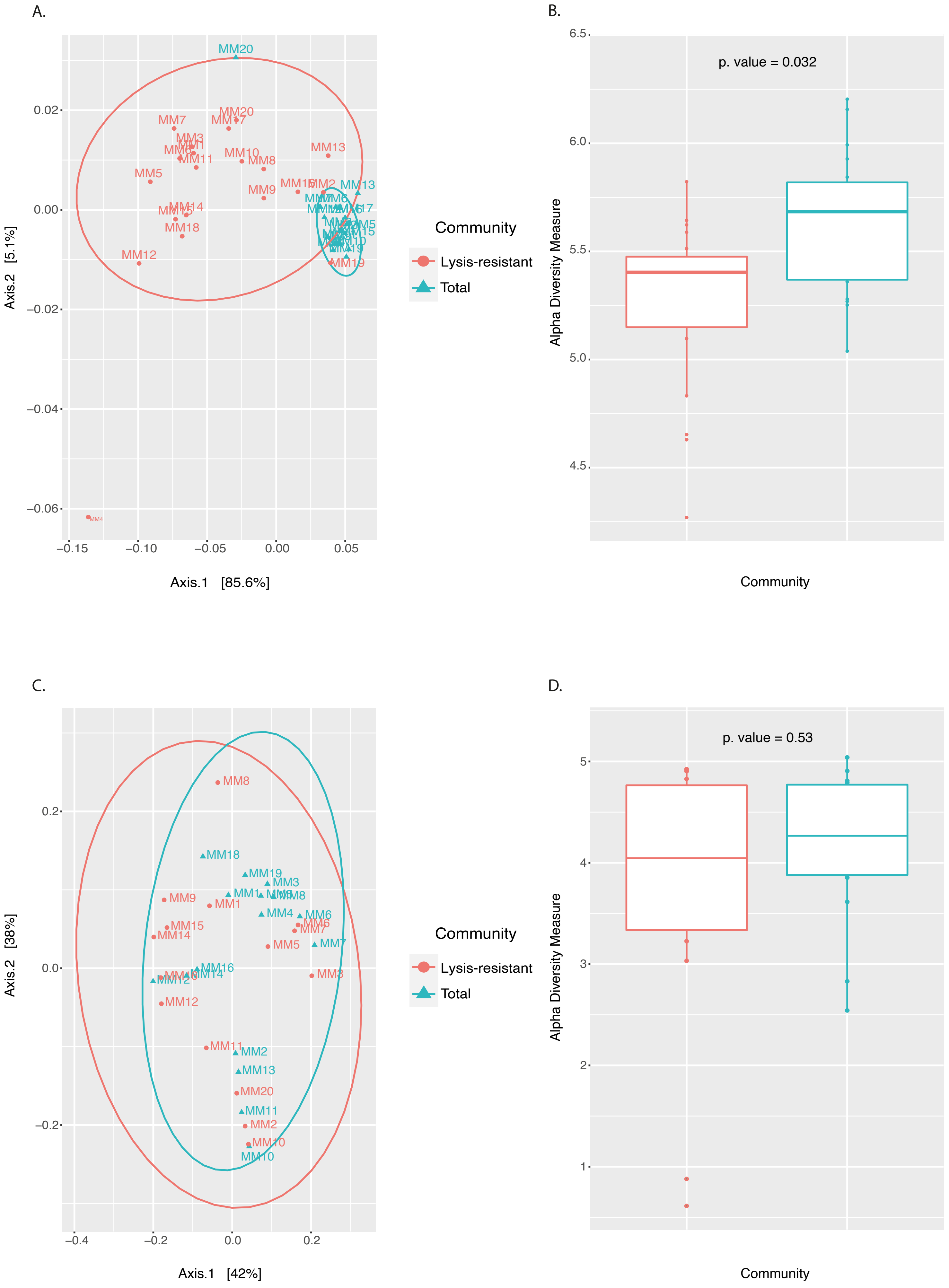

Supplementary Figure 7. Analysis of the microbial mat community **A.** PCoA of the bacterial community. **B.** Alpha diversity of the bacterial community. **C.** PCoA of the archaeal community. **D.** Alpha diversity of the archaeal commu-nity.
