# Supplementary figures and images for: Diversity of Lysis-Resistant Bacteria and Archaea in the Polyextreme Environment of Salar de Huasco
Source: Front Microbiol. 2022 Apr 25;13:826117. doi: 10.3389/fmicb.2022.826117 (PMC9847572; doi:10.3389/fmicb.2022.826117)

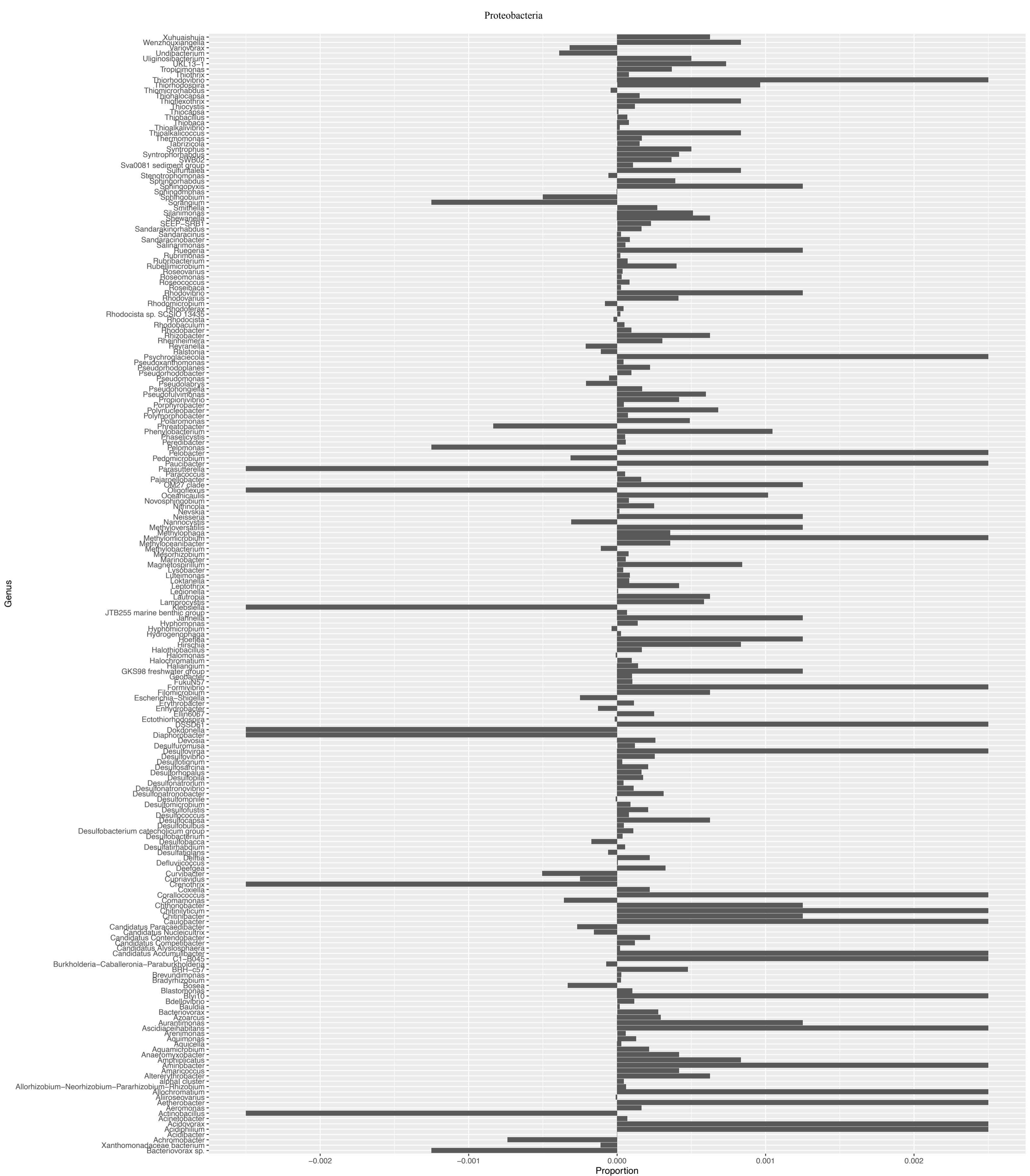

Supplement: Supplementary file 10 [file Data_Sheet_10.PDF]
